# Characterization of Two *Fusarium solani* Species Complex Isolates from the Ambrosia Beetle *Xylosandrus morigerus*

**DOI:** 10.3390/jof8030231

**Published:** 2022-02-26

**Authors:** Nohemí Carreras-Villaseñor, José B. Rodríguez-Haas, Luis A. Martínez-Rodríguez, Alan J. Pérez-Lira, Enrique Ibarra-Laclette, Emanuel Villafán, Ana P. Castillo-Díaz, Luis A. Ibarra-Juárez, Edgar D. Carrillo-Hernández, Diana Sánchez-Rangel

**Affiliations:** 1 Red de Estudios Moleculares Avanzados, Instituto de Ecología A.C., Carretera Antigua a Coatepec 351, Xalapa, Veracruz 91070, Mexico; nohemi.carreras@inecol.mx (N.C.-V.); benjamin.rodriguez@inecol.mx (J.B.R.-H.); luis.alberto.martinez.rodriguez@hotmail.com (L.A.M.-R.); ljosuelira@gmail.com (A.J.P.-L.); enrique.ibarra@inecol.mx (E.I.-L.); emanuel.villafan@inecol.mx (E.V.); anapatriciacastillodiaz@gmail.com (A.P.C.-D.); luis.ibarra@inecol.mx (L.A.I.-J.); edgar.carrillo@posgrado.ecologia.edu.mx (E.D.C.-H.); 2 Cátedra Conacyt en el Instituto de Ecología A.C., Carretera Antigua a Coatepec 351, Xalapa, Veracruz 91070, Mexico

**Keywords:** ambrosia fungi, symbiote fungi, *Fusarium*, *Xylosandrus morigerus*, phytopathogen

## Abstract

Ambrosia beetles are insect vectors of important plant diseases and have been considered as a threat to forest ecosystems, agriculture, and the timber industry. Several factors have been suggested as promoters of the pathogenic behavior of ambrosia beetles; one of them is the nature of the fungal mutualist and its ability to establish an infectious process. In Mexico, *Xylosandrus morigerus* is an invasive ambrosia beetle that damages many agroecosystems. Herein, two different isolates from the *X. morigerus* ambrosia beetle belonging to the *Fusarium* genus are reported. Both isolates belong to the *Fusarium solani* species complex (FSSC) but not to the Ambrosia *Fusarium* clade (AFC). The two closely related *Fusarium* isolates are pathogenic to different forest and agronomic species, and the morphological differences between them and the extracellular protease profile suggest intraspecific variability. This study shows the importance of considering these beetles as vectors of different species of fungal plant pathogens, with some of them even being phylogenetically closely related and having different pathogenic abilities, highlighting the relevance of the fungal mutualist as a factor for the ambrosia complex becoming a pest.

## 1. Introduction

An insect–fungus mutualism is an interaction that implies a reciprocal influence where each species provides mutual benefits such as dispersal, protection and nutrition [1]. The supply of nutrients by a partner can be direct, by serving them as food, or indirect, by providing digestible compounds or detoxifying a food source. They protect each other against environmental variations, competitors and/or natural enemies, and the dispersal aspect is clearly a benefit for the fungus, since it is a sessile organism and uses the insect as vector for its spores or propagules [1]. Fungiculture is the best-known mutualistic interaction between insect and fungi. This activity is performed by fungus-farming ants (Hymenoptera: Formicidae: Myrmicinae: Attini) [2], fungus-farming termites (Blattodea: Termitidae: Macrotermitinae) [3], the stingless bee *Scaptotrigona depilis* (Hymenoptera: Apidae: Meliponini) [4,5] and bark and ambrosia beetles (Coleoptera: Curculionidae: Scolytinae and Platypodinae) [6].

Bark and ambrosia beetles (Curculionidae: Scolytinae) have been adopted as a model of evolutionary ecology and phytopathology since they have emerged as a threat to forests and agricultural areas. Among these beetles, there are 16 hypothesized origins of fungus farming [7,8], with 63 genera in 10 tribes [9], and members of the tribe Xyleborini are considered to be strict fungus farmers, e.g., the genera *Euwallacea*, *Xyleborus*, *Xyleborinus*, and *Xylosandrus* [8]. Ambrosia beetles are xylem-borers of dead and stressed trees and feed primarily on cultivated co-evolved fungi, from which they acquire nutrients such as amino acids, vitamins and sterols; at the same time, fungus grows as mycelium on the walls of their galleries. Most of the ambrosia beetle species transport their food (fungi) in the mycetangium or gut [9,10,11], and it is proposed that the mycetangium may enforce the fidelity to the fungal mutualist [12].

The fungal mutualists of ambrosia beetles are reported to be Ascomycetes—belonging to the orders Ophiostomatales, Microascales, Hypocreales, and Saccharomycetales—and Basidiomycetes, of the orders Russulales and Polypolares [7]. Some species of the genera *Ambrosiella* (Microascales)*, Raffaelea* (Ophiostomatales), *Geosmithia* (Hypocreales) and *Ambrosiozyma* (Saccharomycetales) are the best-known ambrosia fungi [6,7,11]; however, several interactions with *Fusarium* (Hypocreales), a genus that encloses several plant pathogens, have been documented [13].

Until now, the *Fusarium* species, described as a nutritional mycangial mutualist of ambrosia beetles, belongs phylogenetically to the Ambrosia *Fusarium* Clade (AFC), which was first described within the *F. solani* species complex (FSSC) as a monophyletic lineage, and which includes several phylogenetically distinct species [14,15,16,17,18,19]. These species from the AFC are obligate mutualists of *Euwallacea* ambrosia beetles (Coleoptera: Scolytinae) and it has been suggested that at least seven *Euwallacea* species are engaged in this obligate mutualism with at least 16 ambrosia fusaria species [19]. However, there is a constant stream of new reports about *Fusarium* species associated with ambrosia beetle species [20,21,22]. The data from these suggest that the *Fusarium* species could be common mutualists from others closely related species belonging to the Xileborini tribe but is clearly non-exclusive from beetles of the *Euwallacea* genus. For example, some species belonging to FSSC have been isolated from different species of the *Xylosandrus* genus, e.g., *X. germanus* [23], *X. crassiusculus* [24] and *X. compactus* [25,26].

The *Xylosandrus* genus includes at least 54 species, such as *X. morigerus*, *X. crassiusculus*, *X. germanus*, *X. compactus* and *X. curtulus* [27,28], and it has been suggested that members of this genus should be considered potential quarantine pests [29]. In particular, *X. morigerus* is defined as an ecological generalist [29]; thus, it can establish itself in new areas and become invasive, damaging agriculture and/or forestry areas under certain conditions. The damage to new environments, such as orchard and urban landscapes, is due to its capacity to attack live but weakened trees [30] and perform long-term attacks [6,30,31], since *Xylosandrus* is attracted to ethanol, which is produced by affected trees during biotic and abiotic stress, generating a continued infestation [30], and also for its association with phytopathogenic fungi [31,32]. The *Ambrosiella* species is considered to be the main fungal mutualist of *Xylosandrus* spp. [33,34]; however, *Ambrosiella* rarely behaves as a phytopathogen. Nevertheless, the acquisition of fungi from the environment modifies the beetle mycobiome [35] and, through lateral transmission, *Xylosandrus* spp. can associate with plant pathogens.

Analyses are currently underway to determine how the acquired plant pathogen impacts the health of an infested tree. An approach to address this matter is pre-invasion assessment [36], a phenotypical analysis of the symbiotes/mutualists, focusing on the behavior of the pathogen in potential hosts. Here, we report the identity of two fungal isolates from *X. morigerus* captured in Veracruz, Mexico. We determined the pathogenic capacity of both isolates in different possible hosts, giving insights into the complex *Xylosandrus–Fusarium* and its phytosanitary implications in some environments.

Understanding this interaction will increase the knowledge of the ecology of the ambrosia complexes and improve the management strategies designed to prevent and control the damage that they can cause.

## 2. Materials and Methods

### 2.1. Fungal Isolation

INECOL_BM-04 and INECOL_BM-06 were isolated from laboratory-reared ambrosia beetles, *Xylosandrus morigerus.* The beetles used to start the colony were collected in 2015 at Jaguaroundi Ecological Park, a protected natural area located in Coatzacoalcos, Veracruz (N 18.10931, W 94.36044). Ambrosia beetles were captured using ethanol-baited traps similar to those described by [37]. To build these traps, the neck of a two-liter polyethylene terephthalate (PET) plastic bottle was removed and joined with adhesive tape to the neck of another two-liter PET plastic bottle that had a cut frame of 11 × 20 cm on the side. A 50 mL Falcon tube with 96% ethanol was tied to the wall of the upper bottle and a cotton cord served as a wick to release the ethanol. In the lower bottle, moistened paper towels were placed to avoid the dehydration of captured beetles. Five of these traps were hung on trees at 5 PM and removed at 7 AM the next morning.

Female beetles were sorted and placed in an artificial culture media [38] for laboratory rearing and incubated at 26°C in total darkness. After 30 days, the colonies were dissected, and 3 females were collected, mounted and morphologically identified using the taxonomic keys of [39]. Female specimens of *X. morigerus* were surface sterilized by vortexing them twice in a 96% ethanol solution for 1 min; then, 10 *X. morigerus* beetles were aseptically segmented into two parts: head/thorax and abdomen. The surfaces of heads/thorax segments were washed by vortexing in a 1.5 mL microcentrifuge tube with 500 μL of 96% ethanol for 30 s and rinsed three times with sterile distilled water. The beetle tissue was ground with a sterile plastic micropistil in a 1.5 mL microcentrifuge tube containing 50 µL of sterile distilled water. Twenty-five microliters of the suspension was spread on a Petri dish with potato dextrose agar (PDA, 39 g/L Sigma-Aldrich, St. Luis, MO, USA) in duplicate and the cultures were incubated at 28 ± 1 °C in darkness. The colonies that showed differences in morphology were selected and placed individually in Petri dishes containing PDA (Sigma-Aldrich, St. Luis, MO, USA), which were incubated at 28 ± 1 °C in darkness for 14 days. These isolates were purified by single conidial culture in water-agar medium (2% agar) and incubated at 28 ± 1 °C for 1 to 3 days in darkness. A single colony was cultured on PDA (Sigma-Aldrich, St. Luis, MO, USA) and incubated at 28 ± 1 °C for 7 days in darkness. Two morphologically different isolates, INECOL_BM-04 and INECOL_BM-06, were selected for further characterization.

The isolates are maintained in the Internal Collection of the Molecular Biology Laboratory at the Department of Advanced Molecular Studies at Ecology Institute (INECOL).

### 2.2. Culture Conditions

The *Fusarium* spp. isolates INECOL_BM-04 and INECOL_BM-06 were cultured by 5 mm diameter mycelium plugs on PDA (Sigma-Aldrich, St. Luis, MO, USA) at 25 ± 1 °C in darkness.

### 2.3. Molecular Identification

The genomic DNA of axenic colonies of INECOL_BM-04 and INECOL_BM-06 was extracted using the protocol described by [40] with minor modifications. The rRNA cluster, consisting of the internal transcribed spacers (ITS1 and ITS2) and the 5.8S rRNA genes, was amplified using ITS4 and ITS5 primers [41], and the PCR mix for the amplification was prepared following the manufacturer’s protocol of the Platinum^®^ Taq DNA Polymerase High Fidelity (Invitrogen, Waltham, MA, USA, Cat. 11304). Amplification was conducted in a MultiGene^TM^ OptiMax thermocycler with the following conditions: 94 °C for 1 min, followed by 32 cycles of 94 °C for 15 s, 58 °C for 30 s, and 72 °C for 1 min with a final extension step of 72 °C for 5 min. The sequences of the purified PCR product were determined in both directions with the BigDye Terminator v3.1 technology and multicapillary DNA sequencing system AB3730, using the specific primers.

### 2.4. Molecular Phylogenetic Analysis

The phylogenetic analysis was based on four gene fragments: the translation elongation factor-1 alpha (tef1), the internal transcribed spacer region of rDNA (ITS), the 28S large subunit of the rDNA (LSU) and the RNA polymerase second-largest subunit (rpb2). The sequences of the FSSC species were those used in previous studies [42,43,44]. The sequences of the four loci of INECOL_BM-04 and INECOL_BM-06 were retrieved from the unpublished assembled genomes, provided by Ibarra-Laclette and Sánchez-Rangel from the Ecology Institute (INECOL) at Xalapa, Veracruz-Mexico. The sequences of the four markers for INECOL_BM-04 and INECOL_BM-06 isolates were submitted to GenBank with the following accessions: OM455454, OM455455, OM455456, OM455457, OM455458, OM455459, OM455460 and OM455461.

The sequences were manually curated when necessary and aligned with ClustalX [45]. After performing a heuristic trimming with Trimal [46], the four markers were concatenated in a single sequence representative of each species and the best model for molecular evolution was identified for each marker using the corrected Aikaike Information Criterion with PartitionFinder2 [47]. The phylogenetic tree and its clade credibility values were inferred using MrBayes [48] through a Markov chain Monte Carlo (MCMC) analysis of 2 runs over 1 × 10^6^ generations. The resulting phylogenetic tree was visualized and annotated using the Ggtree [49] and the Treeio [50] packages in R. The four-locus data set and the Bayesian Inference (BI) tree are publicly available in TreeBASE (http://purl.org/phylo/treebase/phylows/study/TB2:S29320, accessed on 24 January 2022).

### 2.5. Macroscopic Morphology Examination

Petri dishes with PDA were inoculated with a 5 mm diameter plug taken from the edge of the actively growing one-week-old colony and incubated at 25 ± 1 °C in darkness. Colony morphology was evaluated seven and fourteen days post inoculation (dpi). The photo-documentation was carried out with a Sony Cybershot DSC-W55 camera.

### 2.6. Colony Radial Growth

Each isolate was cultivated in darkness at 25 ± 1 °C in PDA. The diameter of the colony was measured at the end of 14 days. The assay was performed with five technical replicates. A one-way ANOVA with post hoc Tukey HSD test was performed for statistical analysis.

### 2.7. Microscopic Analyses

Microscopic characters were investigated in Spezieller Nährstoffarmer Agar (SNA) and Carnation Leaf Agar (CLA) [51] at 25 ± 1 °C under a photoperiod of 12 h light/12 h dark for 10 days. The presence of sporodochia and chlamydospores was evaluated after one month of incubation. Conidia, conidiophores and chlamydospores were examined and documented with a Leica DMI6000 B microscope using a Leica DFC450 C camera and recorded using LAS X software after they were mounted in water. Average, standard deviation (SD) and minimum–maximum values for the size of individual conidial types for each isolate were calculated from measurements of at least 30 randomly selected conidia. The conidia from SNA cultures were collected with sterile distilled water and quantified in a Leica DM750 microscope using a Neubauer chamber. The sporodochia images were acquired with a Zeiss SteREO Discovery.V8 stereomicroscope. For scanning electron microscopy (SEM), 5 mm mycelium plugs were taken from the actively growing edge of a five-day-old colony cultured on PDA (Sigma-Aldrich, St. Luis, MO, USA) and fixed in 2.5% glutaraldehyde for 24 h at 4 °C and washed three times in 0.1 M Sorensen’s phosphate buffer for 5 min each. The samples were dehydrated through a graded ethanol series (30, 40, 50, 60, 70, 80, 90 and 96%); each sample remained for 60 min in each dilution and was then transferred to absolute ethanol three times (30 min each). Dehydrated samples were dried up to the critical point with CO_2_ in a Polaron-E500 drying apparatus. Dried samples were mounted on aluminum stubs and coated with gold using a Polaron 11-HD sputter-coating unit and observed in an FEI Quanta^TM^ 250 FEG SEM operating at 5 kV [52].

### 2.8. Pathogenesis Assay in Coffea arabica, Salix lasiolepis, Populus nigra, Citrus sinensis and Citrus latifolia

*Coffea arabica* cv. Marsellesa trees were purchased from Sociedad de Productores de Café sustentable Aromas de Coatepec SPR de RL in Emiliano Zapata, Veracruz; this organization acquired the coffee seeds from the germplasm bank of Cafetalera Guadalupe Zajú, S.A de C.V., which is Rainforest Alliance certified. *C. arabica* cv. Oro Azteca trees were kindly donated by Instituto Nacional de Investigaciones Forestales, Agrícolas y Pecuarias (INIFAP). *S. lasiolepis* and *P. nigra* trees were acquired from a Las Palmas nursery in Saltillo, Coahuila, Mexico; this nursery is registered at the Secretaría del Medio Ambiente y Recursos Naturales (UMA-VIV-0605-COA) as a Management Unit for Wildlife Conservation. Meanwhile, *C. sinensis* and *C. latifolia* trees were bought from the “El Olmo” nursery in Xalapa, Veracruz, Mexico. The age of the trees ranged between one and two years and their height varied from 1.40 to 2.20 m. The trees were maintained in a zenith-type greenhouse under the following conditions: minimum temperature of 19 ± 2 °C, maximum of 27 ± 2 °C and a relative humidity between 50 and 70%. Nutrient solution (Nitro Sol) was periodically applied to all the plants to increase vegetative development.

Stem segments 8 cm in length were cut longitudinally and, together with the leaves of each plant species, were disinfected with a 2% (*v/v*) chlorine solution for 2 min and washed three times (2 min each) with sterile distilled water. For the pathogenesis assay, a wet chamber system was used, consisting of a 150 mm × 25 mm Petri dish with a cellulose filter paper (Whatman Grade 1) placed at the bottom and saturated with sterile distilled water. Two to three leaves or stems of the plant species were placed in the wet chamber. Before the inoculation, mechanical damage was inflicted with a scalpel at the base of the leaf and the central part of the stem. The plant tissue was inoculated by placing on the damaged site a plug of mycelium from the edge of the actively growing colony in seven-day-old PDA (the plug was carefully placed so the mycelium touched the plant tissue). The wet chamber was sealed and incubated at 25 °C ± 1 under a photoperiod of 8 h/16 h light/darkness for 12 days and for 21 days in the case of *C. arabica*. The assay was performed with three technical replicates. The lesion area was measured by Image J conducting particle quantification based on image contrast.

### 2.9. Extracellular Protease Activity Assay

The extracellular protease activity of both *Fusarium* sp. isolates was evaluated with a milk powder plate assay in six different growth media: PDA (BD-Difco^TM^), Minimal Medium (MM) (+C+N), MM without nitrogen source (+C−N), MM without carbon source (−C+N), MM without carbon and nitrogen source (−C−N) and water agar 1.5% (WA). MM and its variants were prepared as described by [51]. A 25% solution of dried skimmed milk was prepared separately and added to the culture media to a final concentration of 3%. Plates were inoculated with a plug of mycelium obtained from the edge of an actively growing colony on PDA and incubated for 7 days at 28 °C. To determine the Lysis Index, the diameter of the protease halo was divided by the diameter of the colony. The assay was performed with three technical replicates. The Heat Map was generated using the GraphPad Prism 8 Software.

## 3. Results

### 3.1. Fungal Isolates INECOL_BM-04 and INECOL BM-06 Belong to the FSSC but Not to the AFC

**Figure 1:**
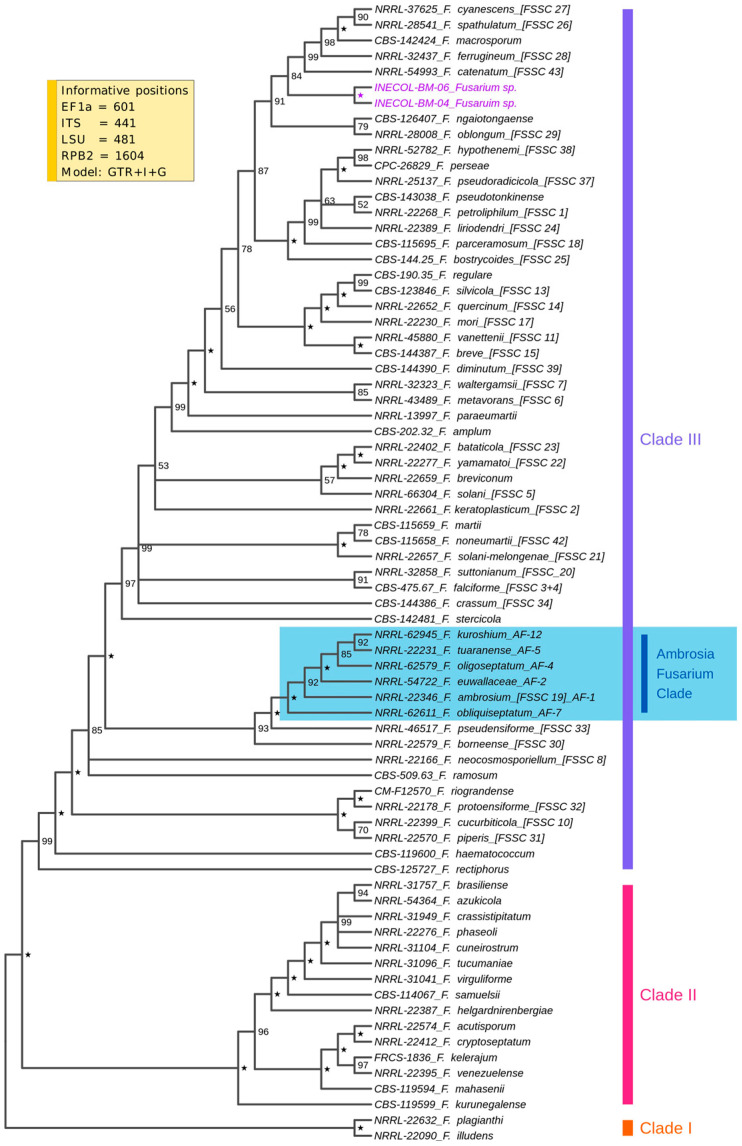
Cladogram of the phylogenetic relationship of *Fusarium* sp. INECOL_BM-04 and *Fusarium* sp. INECOL_BM-06 with members of the three clades of FSSC. Phylogeny constructed through Bayesian inference using a combined data set of four gene markers’ (*tef*1, ITS, LSU and the second-largest subunit of RNA polymerase II (*rpb*2)) sequences. The representative species of clades I, II and III are those proposed by [53]. Numerical designations referring to the informal nomenclature for phylogenetic species of FSSC (e.g., FSSC 1) are provided. The AFC is indicated. Support values from the Bayesian inference are indicated at the nodes. A star in the node indicates 100% support. INECOL isolates are highlighted.

A BLAST analysis of the ITS sequence of both isolates against the ITS database of NCBI showed 96.13% and 96.30% identity to *Fusarium solani* CBS 140079 for INECOL_BM-04 and INECOL_BM-06, respectively. These ITS sequences also compared by BLAST against the Fusarioid-ID database [42] were similar to those of FSSC 12a NRRL 46705, with 98.58% identity for INECOL_BM-04 and 98.75% identity for INECOL_BM-06. Based on the results described above, these isolates were considered as members of the genus *Fusarium* belonging to the FSSC. To improve and establish their phylogenetic relationship with other species from FSSC, a multilocus sequence analysis based on the *tef*1, ITS, LSU and *rpb2* sequences was performed. The Bayesian inferred phylogeny included 3127 bp characters from these four loci and from a total of 73 strains belonging to the three clearly distinguishable subclades from FSSC (Figure 1). The multilocus sequence typing revealed that both INECOL isolates belong to clade 3 but not to AFC, which is made up of *F. ambrosium* AF-1, *F. euwallaceae* AF-2, *F. oligoseptatum* AF-4, *F. tuaranense* AF-5, *F. obliqueseptatum* AF-7 and *F. kuroshium* AF-12. However, both INECOL isolates formed a highly supported clade showing the close relatedness among them and are related to the clade represented by *F. macrosporum* CBS 142424, *F. spathulatum* NRRL 28541 (FSSC 26), *F. cyanescens* CBS 518.82 (FSSC 27), *F. ferrugineum* NRRL 32427 (FSSC 28) and *F. catenatum* NRRL 54993 (FSSC 43).

### 3.2. The Two Closely Related Fusarium sp. INECOL_BM-04 and INECOL_BM-06 Isolated from X. morigerus Are Phenotypically Different

**Figure 2:**
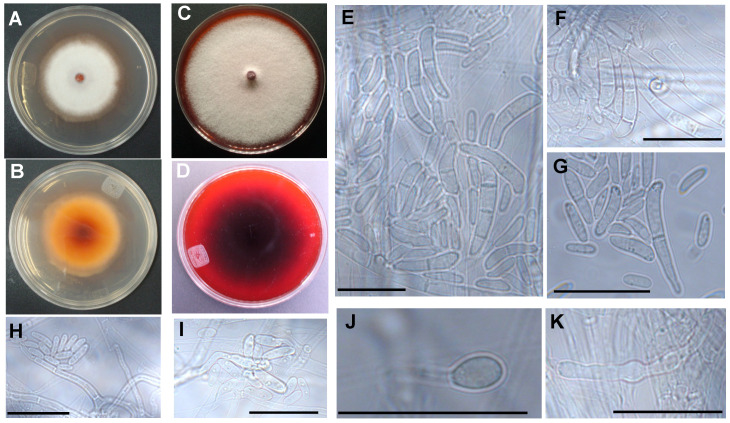

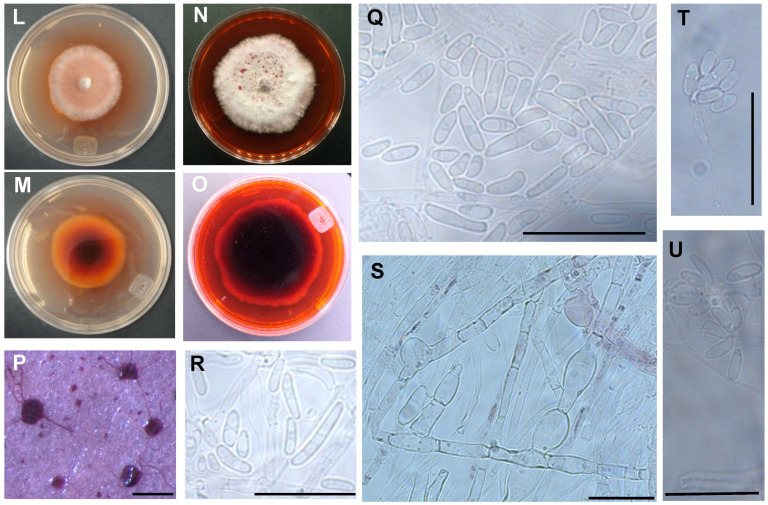
Morphological characteristics of *Fusarium* sp. INECOL_BM-04 and *Fusarium* sp. INECOL BM-06 isolated from *X. morigerus.* Colony morphology in PDA at 7 and 14 days of incubation of *Fusarium* sp. INECOL_BM-04: (**A**,**C**) colony surface; (**B**,**D**) colony undersurface, and *Fusarium* sp. INECOL_BM-06; (**L**,**N**) colony surface; (**M**,**O**) colony undersurface. Microscopic characters of *Fusarium* sp. INECOL_BM-04: conidia (**E**–**G**), chlamydospores (**J**,**K**), and aerial conidiophores (**H**,**I**) in SNA or CLA; and *Fusarium* sp. INECOL_BM-06: sporodochia in PDA (**P**), conidia (**Q**,**R**), chlamydospores (**S**) and aerial conidiophores (**T**,**U**) in SNA or CLA. Photographs were taken at 10 dpi. Scale bar = 25 µm; scale bar in *p* = 1 mm.

*Fusarium* sp. INECOL_BM-04 and INECOL_BM-06 showed differences in the morphology of their colonies. After 7 days of incubation in PDA, *Fusarium* sp. INECOL_BM-04 developed a dense white mycelium and the colony presented a smooth margin (Figure 2A,B). Meanwhile, *Fusarium* sp. INECOL_BM-06 showed aerial white mycelium in the periphery with a lower density in comparison with *Fusarium* sp. INECOL_BM-04. The colony also presented concentric rings of purplish mycelium and secreted a reddish compound shown by the pigmentation of the agar (Figure 2L,M). Both isolates presented reddish pigmentation in the reverse of the colony, although in different amounts (Figure 2B,M). At 14 days of incubation, *Fusarium* sp. INECOL_BM-04 developed a dense cottony mycelium with pale cream-white tonalities. The colony presented a smooth margin, and a strong moldy odor was detected (Figure 2C,D). Meanwhile, *Fusarium* sp. INECOL_BM-06 showed aerial white mycelium with a higher density and irregular margin of the colony in comparison with *Fusarium* sp. INECOL_BM-04. The colony presented reddish exudate droplets and a strong moldy odor (Figure 2N,O). Both isolates secreted an intense violet compound shown by the pigmentation of the agar (Figure 2D,O). The colony radial growth was statistically different since *Fusarium* sp. INECOL_BM-04 and INECOL_BM-06 presented mean colony diameters of 86.62 ± 1.77 mm and 66.51 ± 3.05 mm, respectively, at the end of 14 days of incubation.

**Figure 3:**
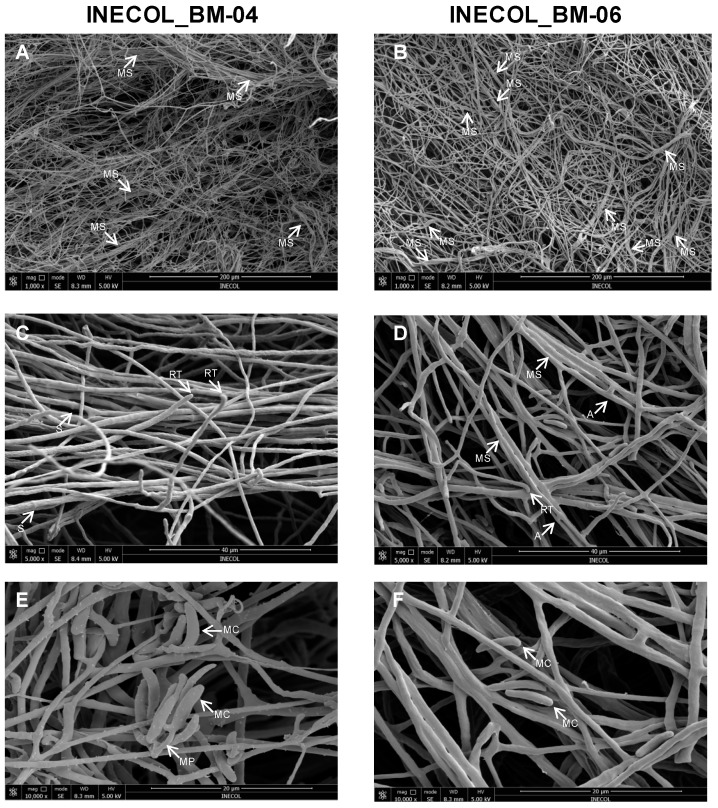
Microscopic features of *Fusarium* sp. INECOL_BM-04 and *Fusarium* sp. INECOL_BM-06, evaluated by scanning electron microscopy (SEM). (**A**,**B**) Micrographs taken at 1000× magnification. (**C**,**D**) Micrographs taken at 5000× magnification. (**E**,**F**) Micrographs taken at 10,000× magnification. MS: myceliar strand; S: septum; RT: rounded tip; A: anastomosis; MP: monophialide; MC: microconidia.

Microscopic characters were evaluated on SNA, CLA and PDA. Both isolates have septate branched hyphae with rounded tips (Figure 3); chlamydospores formed but were scarce, intercalary in or the terminal of the hyphae, mostly globose, single, hyaline, and smooth walled (Figure 2J,K,S). Purple sporodochia were observed in *Fusarium* sp. INECOL-BM-06 (Figure 2P) and absent in *Fusarium* sp. INECOL_BM-04 at 6 weeks of incubation in PDA. Aerial conidiophores were scarce, harboring aseptate microconidia-forming false heads (Figure 2H,I,T,U). There were also conidiophores arising from the substrate mycelium with a simple phialide-forming aseptate microconidia in *Fusarium* sp. INECOL-BM-06 (Figure 2Q,R) and 1–3 septate conidia in *Fusarium* sp. INECOL_BM-04 (Figure 2E–G). Aseptate microconidia were indistinguishable from aerial or substrate mycelium conidiophores. Microconidia from *Fusarium* sp. INECOL-BM-04 were hyaline and obovoid, reniform, oval or allantoid shaped, measuring 6.6 − (9.6 ± 1.8) − 17 × 2.1 − (3.1 ± 0.6) − 5.3 µm; in contrast, *Fusarium* sp. INECOL-BM-06 had hyaline-, obovoid-, oval-, fusiform- and allantoid-shaped and 5.6 − (9.1 ± 1.8) − 20.1 × 2.3 − (3.1 ± 0.4) − 4.2 µm-sized microconidia. The 1-3 septate conidia from *Fusarium* sp. INECOL_BM-04 were scarce in comparison with aseptate conidia and 15.8 − (22.2 ± 5) − 34.3 × 1.1 − (4.3 ± 0.8) − 6.6 µm sized. *Fusarium* sp. INECOL_BM-04 had higher production of conidia in comparison to *Fusarium* sp. INECOL_BM-06, 3.13 × 10^6^ conidia (±1,102,065.94) and 1.39 × 10^6^ conidia (±302,108.148) per colony, respectively. By means of SEM, a myceliar organization was more evident, composed of loosely organized hyphal filaments, hyphal aggregates, such as myceliar strands, and some interconnects by anastomosis (Figure 3); our observations suggest that *Fusarium* sp. INECOL_BM-06 develop a more robust network since the myceliar strands and anastomosis are more evident in this species (Figure 3).

Based on the molecular and morphological characters, these two isolates associated with *Xylosandrus morigerus* can be considered to be closely related, belonging to the *Fusarium* genus, specifically to the FSSC but not to AFC.

### 3.3. Phytopathogenicity Screening Shows Differences in Virulence among Fusarium spp. Associated with Xylosandrus morigerus

To probe whether *Fusarium* spp. isolated from *X. morigerus* exhibits phytopathogenic behavior, we implemented various pathosystems using a wide range of hosts, including agronomical species, principally those that are important in Veracruz, Mexico, such as *Coffea arabica*, which is a known host of *X. morigerus*, and species of *Citrus*, as well as forest species that are potential hosts of *X. morigerus.* Both isolates provoked disease symptoms in all the plant species used; however, we observed important differences between assays and the *Fusarium* isolates.

#### 3.3.1. Pathogenesis Assays in *Coffea arabica*

**Figure 4:**
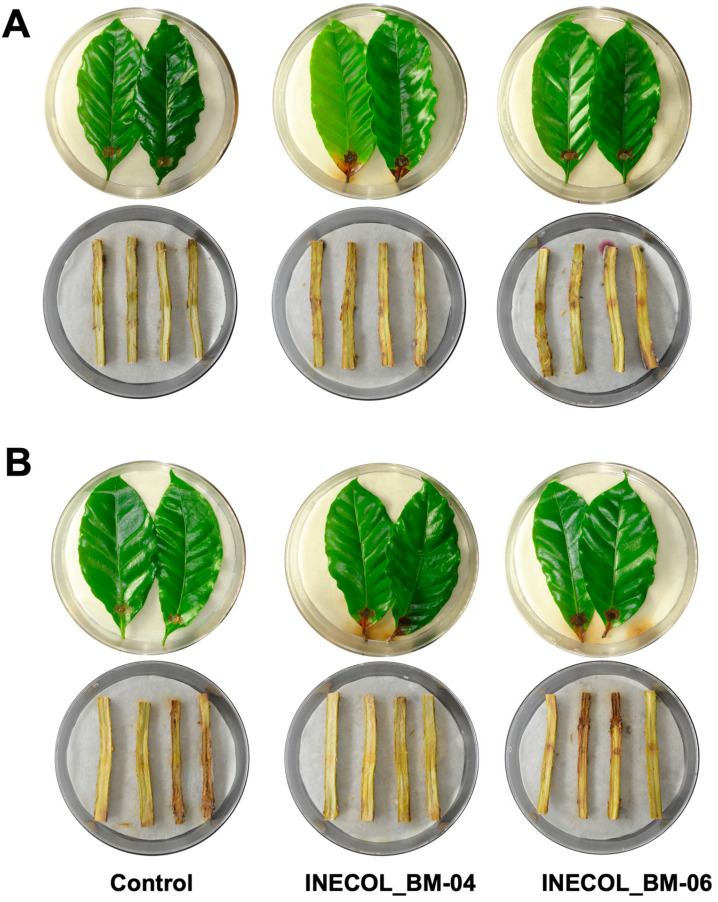
Pathogenicity screening of *Fusarium* sp. INECOL_BM-04 and *Fusarium* sp. INECOL_BM-06 isolates in leaves and vascular tissue of *Coffea arabica*. (**A**) *C. arabica* cv. Marsellesa (**B**) *C. arabica* cv. Oro azteca. Photographs were taken at 21 dpi.

Leaves and stems of *C. arabica* cv. Marsellesa and cv. Oro azteca were challenged with *Fusarium* spp. (Figure 4). Both isolates provoked infection symptoms of necrosis, discreetly; however, the damage in leaves of *C. arabica* cv. Marsellesa inoculated with INECOL_BM-04 was statistically higher in comparison with those inoculated with INECOL_BM-06, with 4.20% (±1.25) and 2.53% (±0.79) of leaf surface being affected by each isolate. Meanwhile, the tissue damage provoked by both isolates in *C. arabica* cv. Oro azteca was similar, *Fusarium* sp. INECOL_BM-04 affected 3.67% (±0.65) and *Fusarium* sp. INECOL_BM-06 damaged 3.35% (±0.70) of the leaf surface (Figure S1). On the other hand, the stems seem to be resistant to *Fusarium* spp., since no differences were detected in comparison with control stems after 21 dpi (Figure 4).

#### 3.3.2. Pathogenesis Assays in Important Citrus Species, *Citrus latifolia* and *Citrus sinensis*

**Figure 5:**
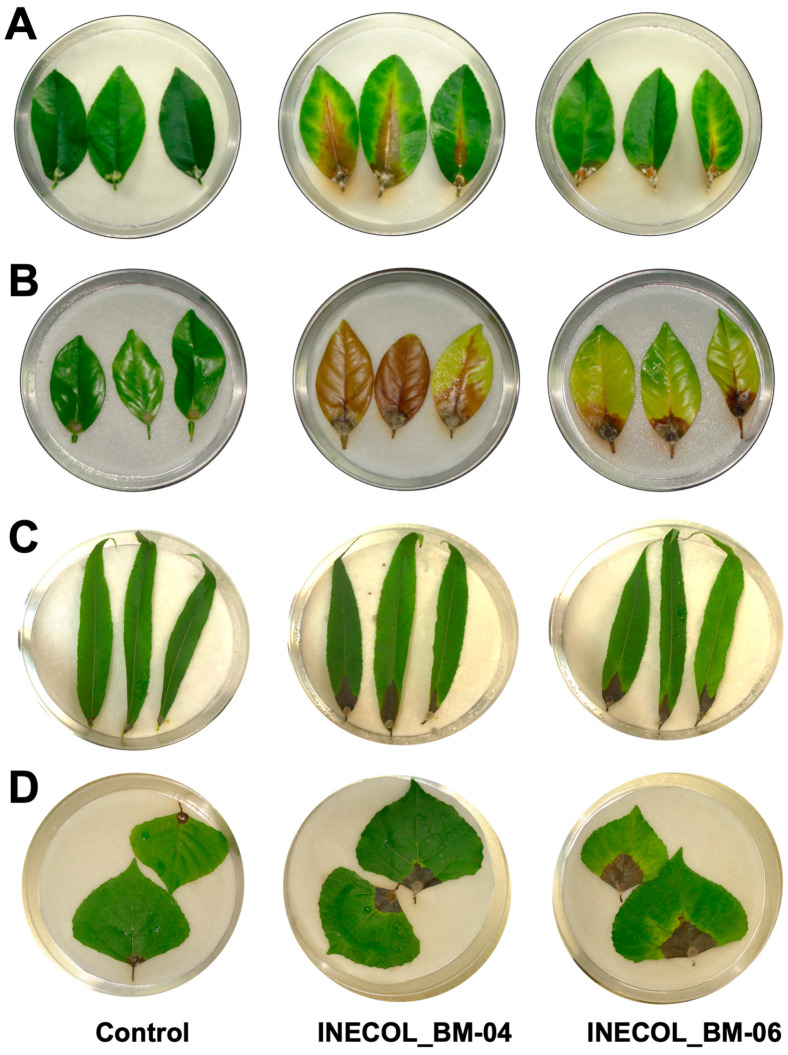
Pathogenicity screening of *Fusarium* sp. INECOL_BM-04 and *Fusarium* sp. INECOL_BM-06 in leaves of citrus and forest species. (**A**) *Citrus latifolia.* (**B**) *Citrus sinensis*. (**C**) *Salix lasiolepis.* (**D**) *Populus nigra.* The photographs were taken at 12 dpi for (**A**–**D**) and 8 dpi for (**B**).

**Figure 6:**
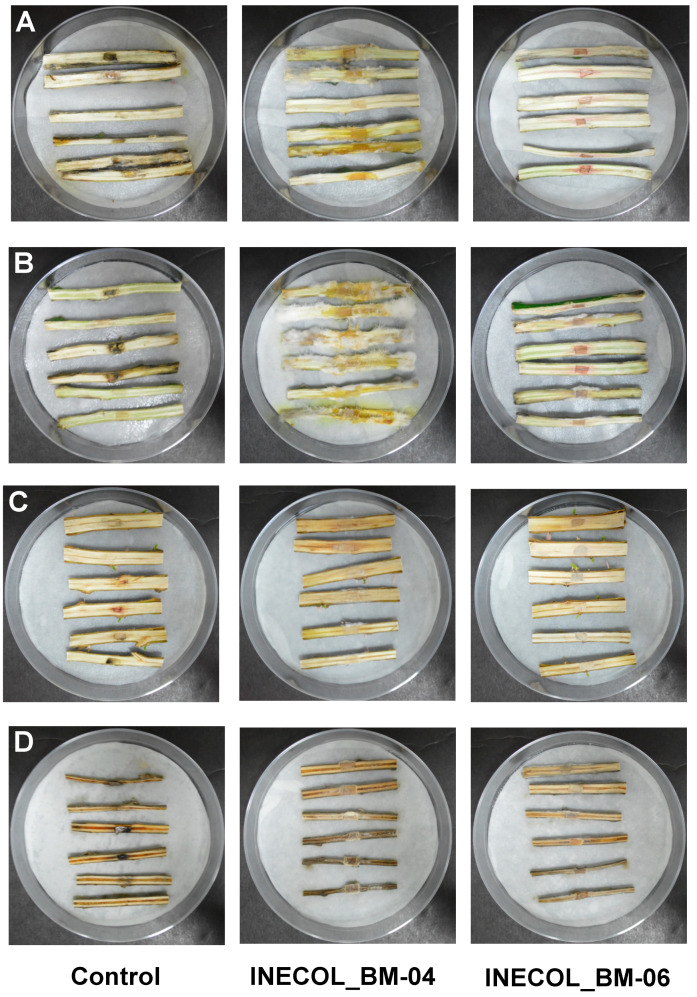
Pathogenicity screening of *Fusarium* sp. INECOL_BM-04 and *Fusarium* sp. INECOL_BM-06 in vascular tissue of citrus and forest species. (**A**) *Citrus latifolia.* (**B**) *Citrus sinensis*. (**C**) *Salix lasiolepis.* (**D**) *Populus nigra.* The photographs were taken at 12 dpi.

In leaves of the agronomical species *C. latifolia* and *C. sinensis*, both isolates triggered clear infection symptoms characterized by chlorosis in the zone of the principal vein and brownish necrosis at 12 dpi. *Citrus sinensis* is more susceptible to *Fusarium* spp. associated with *X. morigerus* infection since the symptoms were more pronounced in this citrus species (Figure 5A,B). Interestingly, in *C. latifolia*, the infection by *Fusarium* sp. INECOL_BM-04 progressed significantly faster than the infection by *Fusarium* sp. INECOL_BM-06, and the expansion of tissue damage triggered by *Fusarium* sp. INECOL_BM-04 was approximately four times more than that damage caused by *Fusarium* sp. INECOL_BM-06 (Figure S1). In *C. sinensis*, no significant differences were observed in the expansion of tissue damage (Figure S1), but the intensity of symptoms differed; the leaves inoculated with *Fusarium* sp. INECOL_BM-04 show a bigger necrosed area than those inoculated with *Fusarium* sp. INECOL-BM-06; however, they present chlorosis, also considered a symptom of infection (Figure 5A,B). Regarding the infection of stems, only *Fusarium* sp. INECOL_BM-04 caused rot, indicated by an amber discoloration; this symptom was more pronounced in *C. sinensis*. Additionally, we clearly observed the presence of white cottony mycelium over the stem tissue. *Fusarium* sp. INECOL_BM-06 did not produce a significant fungal mass nor disease symptoms in both citrus species (Figure 6A,B).

#### 3.3.3. Pathogenesis Assays of *Salix lasiolepis* and *Populus nigra*

The pathogenicity tests in leaves of *S. lasiolepis* and *P. nigra* exhibited differences in both pathogenicity and host susceptibility (Figure 5C,D). In *S. lasiolepis*, both isolates provoked statistically similar effects as the mean percentage of the tissue damage was 13% (±2.73 for *Fusarium* sp. INECOL-BM-04 and ±2.46 for *Fusarium* sp. INECOL-BM-06) (Figure S1) and the main symptom was necrosis in the inoculation zone and beyond (Figure 5C). In contrast, for *P. nigra*, a statistically significant difference between the pathogenicity of both isolates was noticed, as *Fusarium* sp. INECOL-BM-06 triggered 19.04% (±5.85) tissue damage in comparison with the 7.30% (±2.13) tissue damage provoked by *Fusarium* sp. INECOL_BM-04 (Figure S1). The necrosis area was accompanied by an accentuated chlorosis in the *P. nigra* leaves infected with *Fusarium* sp. INECOL_BM-06; on the other hand, the leaves infected with *Fusarium* sp. INECOL_BM-04 did not show evident chlorosis symptoms (Figure 5D). With respect to the vascular tissue infection, in both plants there was a development of mycelium (Figure 6C,D); the inoculated stems of *S. lasiolepis* and *P. nigra* showed slight necrosis in vascularity in comparison with their respective controls, being accentuated in those inoculated with *Fusarium* sp. INECOL_BM-04 (Figure 6C,D); however, there are no significant differences in tissue damage.

### 3.4. Extracellular Protease Activity Is Slightly Different among Fusarium spp. Associated with Xylosandrus morigerus

**Figure 7:**
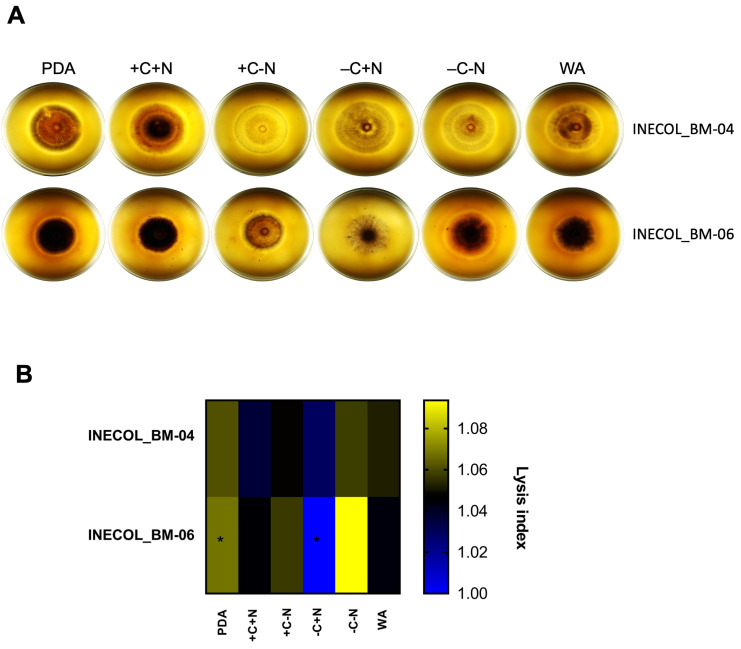
Extracellular Protease activity of *Fusarium* sp. INECOL_BM-04 and *Fusarium* sp. INECOL_BM-06. (**A**) Protease activity of *Fusarium* spp. associated with *X. morigerus* indicated by clearing zones in different culture media supplemented with low-fat powder milk 3%. (**B**) Heat Map of Protease secretion index (normalized by colony diameter) of *Fusarium* spp. associated with *X. morigerus* grown in different culture media supplemented with low-fat powder milk 3%. Heat Map shows the average of three technical replicates. Data obtained 7 dpi. (* *p*-value < 0.05 obtained by one-way ANOVA with Bonferroni multiple comparison calculation). Lysis index = 1 indicates no Lysis.

Secreted proteases contribute to fungal virulence either for the degradation of host tissue, facilitating penetration, or for the acquisition of nutrients. Protease secretion, observed as a lysis halo in the plates, was evaluated and compared in different nutritional conditions in both *Fusarium* isolates associated with *X. morigerus* (Figure 7A). *Fusarium* sp. INECOL_BM-04 secretes proteases in all the tested conditions and the quantities of secreted proteases seem to be similar; however, it secretes higher amounts of proteases in the C-rich condition (PDA) and in the absence of C and N sources (−C−N) (Figure 7B);. A smaller amount of secreted proteases was found in both culture medium with the presence of an N source, +C+N and −C+N (Figure 7B). In contrast, *Fusarium* sp. INECOL_BM-06 is clearly the null lysis halo in the absence of a C source (−C+N) compared with the rest of conditions (Figure 7A,B); thus, growing in the −C+N condition has a repressive effect on protease secretion in this species, while the absence of both C and N sources (−C−N) and the C-rich medium PDA have an inductive effect, followed by the +C−N condition (Figure 7A,B). For both isolates, higher amounts of secreted proteases were observed when there was no N source.

## 4. Discussion

Bark and ambrosia beetles have emerged as study models in phytopathology, since invasive species have changed their behavior by attacking living and healthy trees, provoking serious phytosanitary problems [30,31,32,54]. In addition, many examples of tree diseases provoked by the fungal mutualist are reported [31,32]. Therefore, it is important to gain a comprehensive understanding of the contribution of the associated fungi, in terms of their pathogenicity, for the development of plant disease.

*Xilosandrus morigerus* should be considered as a potential quarantine pest; its introduction and establishment in non-native habitats are facilitated by its regular inbreeding, males mating with their sisters within the parental gallery before dispersal; haplodiploidy, females that are not inseminated by a brother before leaving the nest can mate with a haploid son produced by an unfertilized egg; and by a broad host range stimulated by their mutualist fungus [29,55]. *Xilosandrus morigerus* has a nearly pantropical distribution. In Latin America, its distribution stretches from Veracruz, Mexico, to Brazil; this ambrosia beetle is able to attack a wide variety of host plants, and therefore it is considered as an important pest of crop and ornamental trees. It is called a “brown coffee twig borer” since it is a well-known pest of *Coffea canephora* and *C. arabica* but it can also attack *Persea americana*, *Theobroma cacao*, and *Salix humboldtiana*, among others [55].

For this work, specimens of laboratory-reared *X. morigerus* were used. Originally, the beetles were captured in the nature reserve Jaguaroundi Park for wildlife conservation in Veracruz State, Mexico. The phylogenetic analysis, based on four genetic markers, ITS, LSU, *tef*1 and *rpb*2, showed that the *Fusarium* spp. associated with *X. morigerus* belong to the FSSC and not to the AFC. The fact that INECOL isolates form an independent clade suggests that they can be classified as conspecific isolates of a new *Fusarium* species within FSSC associated with *X. morigerus;* however, future work needs to be carried out in order to define new species and it will be interesting analyze a higher number of isolates to evaluate the extent of genetic variation within the species [56,57].

*Fusarium* spp. associated with *X. morigerus* INECOL_BM-04 and –06 are phylogenetically related to *Fusarium* species, which are little described and morphologically different. Only *F. macrosporum* CBS 142424 is related to plants since it was isolated from *C. sinensis* crown; the rest are related to animals. *F. spathulatum* NRRL 28541 (FSSC 26), *F. cyanescens* CBS 518.82 (FSSC 27) and *F. ferrugineum* NRRL 32427 (FSSC 28) were isolated from human tissues; *F. catenatum* NRRL 54993 (FSSC 43) was isolated from *Stegostoma fasciatum* (zebra shark) tissues [58]. *Fusarium* species have been identified as symbiotes of *Xylosandrus* species and pathogenic for the plant host based on pathogenic tests or because the recovery of the fungus was from infected plant tissue. In Hawaii, *X. compactus* inoculated *F. solani* in *Acacia koa* but virulence assays showed that *F. solani* was not pathogenic [59,60,61]. In Italy, *F. solani* was isolated from entry holes, galleries and stained woody tissues of *Quercus ilex* attacked by *X. compactus*, and *a* pathogenicity test showed the symptoms exhibited by the naturally infected plants [62]; in a National Park in Lazio, *X. crassiusculus* was trapped when attacking *Ceratonia siliqua*, and *F. solani* was isolated from necrotic tissue observed in the proximity of the entry holes [24]. In addition, *Fusarium* spp. were found to be associated with *X. compactus* in the Mediterranean maquis [63]. In New York apple orchards, *X. germanus* was claimed to be the agent that caused damage; *F. solani* was isolated from unsterilized insect bodies, internal contents and infested wood surrounding the entry hole, but the authors attributed the wilting symptoms observed in the trees to the tunneling of the insects [23]. Interestingly, our results suggest that other *Fusarium* species, especially those belonging to FSSC, aside from *F. solani* could be mutualists of *Xylosandrus* spp.

Our analyses showed that even *Fusarium* spp. associated with *X. morigerus* INECOL_BM-04 and –06 are closely genetically related isolates, and they exhibit a particular pathogenic profile and specific morphological traits; these differences can be explained by intraspecific trait variation that can be driven at the gene expression regulation level in response to specific conditions [64,65]. The possible variances in gene regulation can impact how the pathogen responds to a specific host and reprograms the expression of its pathogenic arsenal, i.e., secreted proteases, toxins, and secondary metabolites, among others. Here, we compared the extracellular protease activity of both *Fusarium* isolates associated with *X. morigerus*. Specifically, the nutrition status was shown to regulate the expression of secreted proteases. For both isolates, higher amounts of secreted proteases were found in the absence of an N source. However, nutrition heterogeneity, as denoted by the profiles of protease activity among them, can impact the virulence in a species-, strain- and host-dependent manner, as reported for *Aspergillus nidulans* and others [66,67,68]. On the other hand, it is interesting that some phenotypical differences are evident in habitual culture conditions such as PDA, which suggests that some regulatory differences among the strains are sharp and do not need special conditions highlight; thus, the gene expression could be regulated differentially in both strains “normally”, and this has repercussions on the phenotype.

As part of an initial approach of pre-invasion assessment, we analyzed the pathogenic behavior of *Fusarium* sp. INECOL_BM-04 and -06 in the leaves and stems of agricultural and ecological representative species. The pathogenicity assays suggest that both isolates are pathogenic for most of the plant species tested; however, the extent of the lesion for most of the leaves was less than 20% of the leaf surface and there was no greater damage in the vascular tissue. Both cultivars of *C. arabica*, were agronomically selected for their high yield and resistance to coffee rust, which can contribute to their resistance to *Fusarium* spp. Meanwhile, the damage in *S. lasiolepis* and *P. nigra* was higher, showing mayor susceptibility. Nevertheless, the results suggest that both isolates of *Fusarium* associated with *X. morigerus* do not represent a threat to *C. arabica*, *S. lasiolepis* and *P. nigra*, but both isolates, especially *Fusarium* sp. INECOL_BM-04 can be considered as minor pathogens for *Citrus* species, given the damaged surface of the leaves and the colonization of the vascular tissue. Interestingly, most of the fungal associates of ambrosia beetles displayed no significant impact on the host during a pre-invasion assessment assay [36] but the possibility of the fungus being more virulent in other conditions was not tested, and so cannot be disregarded.

Traditionally, it has been considered that an ambrosia beetle has a single, primary fungal mutualist, but now it is known that most ambrosia beetles carry, and probably feed on, multiple fungal species [6,69]. Additionally, vector shifts have been reported for some fungal mutualist/symbiotes. In addition to *X. glabratus*, *Raffaelea lauricola* was recovered from other ambrosia beetle species, including *X. crassiusculus* [70]. Even, *Fusarium* spp. has been associated with other insects, beyond *Xylosandrus* spp. and *Euwallacea* spp., and with the decline of forest and agricultural species [71,72]. Given these dynamic associations between beetles and fungal mutualists and varying fidelity, studies to determine the mycobiota of bark and ambrosia beetles, by DNA metabarcoding, have been conducted [20,21,35,73,74]. Altogether, these data show the high diversity of fungal–beetle associations and the probability of being a vector of phytopathogens in diverse ecological niches, even without being the main fungal mutualist. In addition to the factors stated before, the variation in trade level among world regions [75] and the influence of spatial and climatic factors in the abundance and dispersal behavior of invasive ambrosia beetle [76] highlight the relevance of studying ambrosia complexes to determine their potential as forest and agricultural threats and, therefore, the implementation of permanent control and surveillance measures worldwide.

## 5. Conclusions

In this study, we report the molecular identification and phenotypical characterization of two fungal isolates from the ambrosia beetle *Xylosandrus morigerus.* The isolates INECOL_BM-04 and INECOL-BM-06 are members of the *Fusarium* genus, belonging to the *Fusarium solani* species complex (FSSC) but not to the Ambrosia Fusarium Clade (AFC), with pathogenic potential with regard to forest and agricultural species. The results highlight the possibility of *X. morigerus* being a vector of phytopathogens.

## Data Availability

Sequences of the four markers for INECOL_BM-04 and INECOL_BM-06 isolates were submitted to GenBank with the following accessions: OM455454, OM455455, OM455456, OM455457, OM455458, OM455459, OM455460 and OM455461. The four-locus data set and the Bayesian Inference (BI) tree are public available in TreeBASE (http://purl.org/phylo/treebase/phylows/study/TB2:S29320?x-access-code=7d6edd9c73c50b876654dd26dec6e00e&format=html, accessed on 24 January 2022).

## Supplementary Materials

The following supporting information can be downloaded at: https://www.mdpi.com/article/10.3390/jof8030231/s1, Figure S1: Percentage of tissue damage in leaves of *Coffea arabica*, *Citrus latifolia*, *Citrus sinensis*, *Salix lasiolepis* and *Populus nigra* inoculated with *Fusarium* sp. INECOL_BM-04 and *Fusarium* sp. INECOL_BM-06.
