# Supplementary material for: Characterization of Two Fusarium solani Species Complex Isolates from the Ambrosia Beetle Xylosandrus morigerus
Source: J Fungi (Basel). 2022 Feb 26;8(3):231. doi: 10.3390/jof8030231 (PMC8956061; doi:10.3390/jof8030231)
Supplement: Supplementary file 1 [file jof-08-00231-s001.zip › jof-1597132-supplementary.pdf]

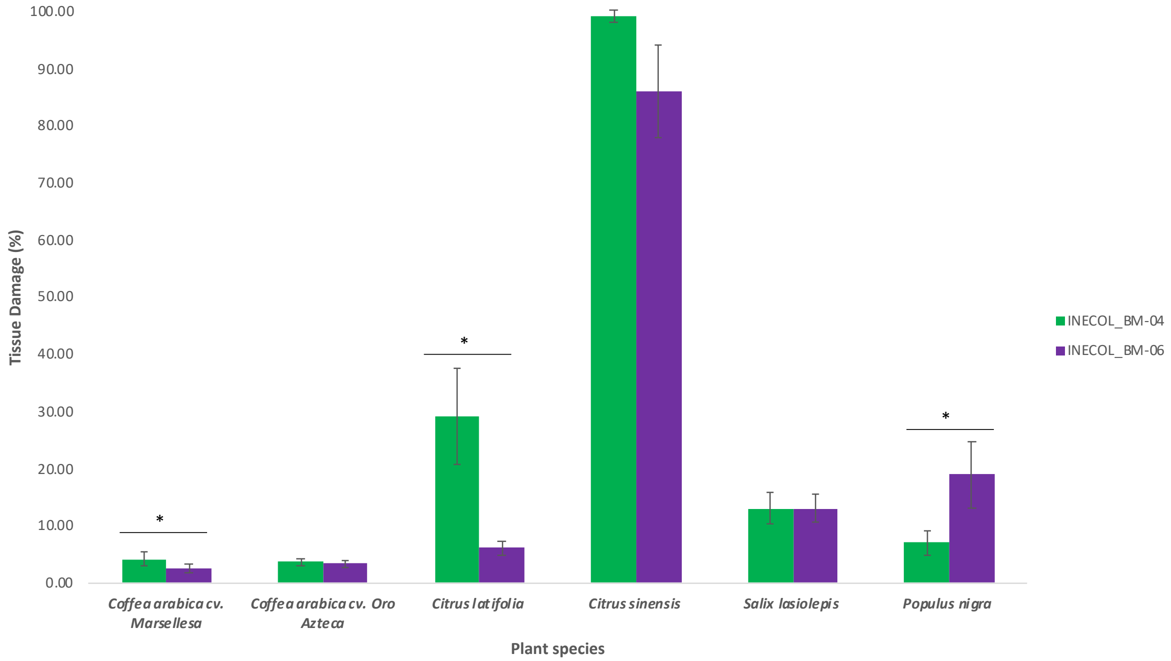

Figure S1. Percentage of tissue damage in leaves of *Coffea arabica, Citrus latifolia, Citrus sinensis, Salix lasiolepis* and *Populus nigra* inoculated with *Fusarium* sp. INECOL_BM-04 and *Fusarium* sp. INECOL_BM-06. The data are the average ±SD (n=9 for *C. latifolia, C. sinensis, S.lasiolepis*, n=6 for *P. nigra* and *C. arabica*). (*p-value<0.05 obtained by One-way Anova with post-hoc Tukey HSD test).
